# Alexithymia, anger and psychological distress in patients with myofascial pain: a case-control study

**DOI:** 10.3389/fpsyg.2013.00490

**Published:** 2013-07-31

**Authors:** Lorys Castelli, Federica De Santis, Ilaria De Giorgi, Andrea Deregibus, Valentina Tesio, Paolo Leombruni, Antonella Granieri, Cesare Debernardi, Riccardo Torta

**Affiliations:** ^1^Department of Psychology, University of TurinTurin, Italy; ^2^Clinical Psychology and Psycho-Oncology Unit, Department of Neuroscience, Health and Science City Hospital, University of TurinTurin, Italy; ^3^Department of Surgical Science, Orthognatodontic and Gnatology Section – Dental School, Health and Science City Hospital, University of TurinTurin, Italy

**Keywords:** myofascial facial pain, anxiety, depression, anger, alexithymia

## Abstract

**Aims:** The aim of this study was to investigate psychological distress, anger and alexithymia in a group of patients affected by myofascial pain (MP) in the facial region.

**Methods:** 45 MP patients [mean (*SD*) age: 38.9 (11.6)] and 45 female healthy controls [mean (*SD*) age: 37.8 (13.7)] were assessed medically and psychologically. The medically evaluation consisted of muscle palpation of the pericranial and cervical muscles. The psychological evaluation included the assessment of depression (Beck Depression Inventory—short form), anxiety [State-Trait Anxiety Inventory Form Y (STAI-Y)], emotional distress [Distress Thermometer (DT)], anger [State-Trait Anger Expression Inventory—2 (STAXI-2)], and alexithymia [Toronto Alexithymia Scale (TAS)].

**Results:** the MP patients showed significantly higher scores in the depression, anxiety and emotional distress inventories. With regard to anger, only the Anger Expression-In scale showed a significant difference between the groups, with higher scores for the MP patients. In addition, the MP patients showed significantly higher alexithymic scores, in particular in the Difficulty in identifying feelings (F1) subscale of the TAS-20. Alexithymia was positively correlated with the Anger Expression-In scale. Both anger and alexithymia showed significant positive correlations with anxiety scores, but only anger was positively correlated with depression.

**Conclusion:** A higher prevalence of depressive and anxiety symptoms associated with a higher prevalence of alexithymia and expression-in modality to cope with anger was found in the MP patients. Because the presence of such psychological aspects could contribute to generate or exacerbate the suffering of these patients, our results highlight the need to include accurate investigation of psychological aspects in MP patients in normal clinical practice in order to allow clinicians to carry out more efficacious management and treatment strategies.

## Introduction

Temporomandibular disorders (TMDs) are a heterogeneous group of disorders associated with dysfunctions of the muscles of mastication, temporomandibular joints (TMJ) and related structures. One of commonest types of TMDs are myofascial pain syndromes (MPS). MPS are characterized by a particular kind of non-articular musculoskeletal pain, associated with discrete taut bands of hardened muscle that contain regions of exquisite muscle dysfunction and tenderness (Travell and Simons, 1999; Shah et al., 2008). The prevalence of MPS ranges between 8 and 20% (Rantala et al., 2003; Janal et al., 2008). Pain in the temporomandibular regions is universally found to be more prevalent in women than in man: it occurs about twice in women than in men. All the studies that assessed its age-specific prevalence rates found that temporomandibular pain is most prevalent in young and middle-aged adults, and declines in frequency among the elderly (LeResche, 1997).

The exact pathophysiology of myofascial pain (MP) is still unclear. Recent studies evidence a multifactorial etiology: genetic, physical, and psychological/psychiatric factors, as well as life-span psychosocial stressors seem to be involved in the onset and evolution of MPS (Kafas and Leeson, 2006; Cairns, 2010).

In MPS, pain is often associated with other symptoms such as depression, chronic stress, anxiety and, consequently, everyday disability (Mongini et al., 2007). Psychological disorders are more common in patients with MPS than in those with other TMDs and the literature show that a relevant percentage of MP patients experience some kind of psychological distress (Korszun et al., 1996; Madland et al., 2000; Celić et al., 2006; Mongini et al., 2007; Giannakopoulos et al., 2010). A high prevalence of depressive symptoms in MP patients, ranging from 23 to 54%, has been detected by many studies (Vimpari et al., 1995; Korszun et al., 1996; Madland et al., 2000; Altindag et al., 2008; Giannakopoulos et al., 2010), whiles the evidence concerning the prevalence of anxiety symptoms in chronic TMDs is still controversial (Manfredini et al., 2004; Mongini et al., 2007; Vedolin et al., 2009; Giannakopoulos et al., 2010). A recent study (Giannakopoulos et al., 2010) assesses anxiety and depression symptoms in chronic temporomandibular pain patients and showed that depression was significantly higher in patients than in controls, while no differences were present for anxiety symptoms. These results agree with the conclusions reached in some previous studies on anxiety (Kino et al., 2001, 2005), whilst other studies have suggested that anxiety plays an important role in temporomandibular pain and increases the likelihood of muscle tenderness (Madland et al., 2000; Manfredini et al., 2004; Mongini et al., 2007). Such contrasting results could be due to methodological issues, for example, the lack of a healthy control group or the use of different and non-specific anxiety assessment instruments.

The role of psychosocial stressors in TMDs has been highlighted in many studies (Tsai et al., 2002; Uhac et al., 2003). Glaros and colleagues studied the role of emotions and stress in predicting facial pain (Glaros et al., 2005). They found that both muscle tension and emotional states were strongly related to pain and suggested that parafunctions could represent an adaptive response to stress (Glaros et al., 2005).

Anger is a multifactorial construct recognized as common among chronic pain patients (Okifuji et al., 1999; Sayar et al., 2004). Anger has often been associated with pain intensity in different pain disturbances, such as fibromyalgia, headache, and regional pain syndrome (Okifuji et al., 1999; Sayar et al., 2004; Trost et al., 2012). To date, no studies have analyzed the role of anger in MPS and only one study has shown a greater anger reaction in response to stressors in patients with TMDs compared with control subjects (Curran et al., 1996).

Alexithymia is an emotional dysregulation trait characterized by difficulties in identifying and describing emotions, tendency to minimize emotional experience and to focus attention externally. Alexithymia is thought to impede successful regulation of emotions, particularly negative affects, resulting in chronic sympathetic hyperarousal, physiological sensations, somatosensory amplifications and physical symptoms complaints (Lumley et al., 2002). The role of the alexithymic trait is increasingly recognized in pain syndromes. Previous studies showed that from a third to 53% of patients with various types of persistent pain appear to be alexithymic (Lumley et al., 1997, 2002; Sayar et al., 2004; Celikel and Saatcioglu, 2006). The association between alexithymia and TMDs has been investigated in only a few studies, with data showing contrasting results (Sipilä et al., 2001; Ahlberg et al., 2004; Glaros and Lumley, 2005). In particular, to date only one study has investigated the presence of alexithymia in MP patients, showing a prevalence of 32.5% (Lumley et al., 2002).

To sum up, while depressive symptoms have been extensively investigated, to date few and contrasting results are available regarding anxiety, alexithymia, and anger in MPS. On this basis, the aim of our study was to investigate alexithymia, anger, and psychological distress in a group of patients affected by MP, by means of a comparison with a control group of healthy women.

## Materials and methods

### Patients

The study was carried out on a sample of 45 consecutive MP patients referred, the first time, to the Gnathological Unit of the Dental School of the “Città della Salute e della Scienza” of Turin (University of Turin). Because the majority of patients who seek treatment for MP are women (LeResche, 1997; Halpern et al., 2007), only female patients with chronic myofascial facial pain (at least 6 months) were included in the study. In addition, because MP declines in frequency among the elderly, we excluded all patients more than 65 years of age. The diagnosis of myofascial facial pain was made according to the guidelines of the American Academy of Orofacial Pain (de Leeuw, 2008). The exclusion criteria were male sex, less than 20 or more than 65 years of age, low educational level (<5 years' schooling), the presence of joint disorders, migraine, neurological disorders, and a history of drug addiction. The final sample had a mean (*SD*) age of 38.9 (11.6) and a mean (*SD*) of educational level of 13 (3.1) years (Table 1).

**Table 1:** **Demographic and clinical characteristics of myofascial pain patients and controls**.

	**Patients**	**Controls**	***T (*df*)***	***P*-value**
Age	38.9 (11.6)	37.8 (13.7)	0.42 (85.6)	0.678
Education	13.0 (3.1)	14.4 (4)	−1.76 (83.4)	0.082
AO	43.2 (5.8)	47.8 (4.5)	−4.14 (88)	<0.001
PO	45.4 (5.4)	49.4 (4.5)	−3.78 (85)	<0.001
PR	5.7 (2.1)	5.9 (1.7)	−0.44 (88)	0.661
LR	7.4 (1.8)	8.2 (2.1)	−1.96 (88)	0.053
LL	7.9 (2.2)	8.6 (2.2)	−1.42 (88)	0.161
PTS	2.0 (0.5)	0.6 (0.5)	14.16 (88)	<0.001
CTS	1.8 (0.7)	0.5 (0.4)	9.86 (67.9)	<0.001

Results of the T-test, used to compare demographic and clinical variable between MP patients and controls. Age and Education are expressed in years. AO, active mouth opening; PO, passive mouth opening; PR, protrusive; LR, lateral right; LL, lateral left; are expressed in mm. PTS, pericranial tenderness score; CTS, cranial tenderness score.

The control group (C group) was composed by a sample of 45 voluntary healthy women, balanced for age [37.8 (13.7) years] and educational level [14.4 (4) years]. The exclusion criteria were: low educational level (<5 years' schooling), the inability to fill out the questionnaires due to an insufficient knowledge of Italian and the presence of organic pathologies, including TMJ disorders, chronic pain, or psychiatric disorders (Axis I and II of the DSM-IV-TR). Written informed consent was obtained from all participants.

### Procedures

Before the medical examination, all the patients and healthy controls filled in a data sheet assessing their medical and psychiatric case history. Medical history included a record of pain characteristics, improvement, or aggravating factors and associated symptoms. All the patients and control subjects were assessed by the same dentist (D.I.).

The psychological scales and inventories were delivered and scored by the same expert psychologist (D.F.), after excluding major mood or anxiety disorders (according to the criteria of the DSM-IV-TR).

### Materials

#### Medical evaluation

Clinical examination consisted of muscle palpation (2 kg/cm^2^ of force) of the pericranial and cervical muscles and was carried out by an expert clinician. The muscles examined were: (1) masseter, (2) lateral pterygoid, (3) medial pterygoid, temporal (4) mandibular, and (5) cranial insertion (together composing the Pericranial Tenderness Score—PTS); sternocleidomastoid (1) belly and (2) cranial insertion, (3) trapetius, (4) nucal muscles (composing the Cranial Tenderness Score—CTS) (Mongini et al., 2007). For each location, tenderness at palpation was scored from 0 to 3, with 0 indicating pressure, 1 bother, 2 pain, and 3 severe pain. Active (AO) and passive (PO) mouth opening (recorded in mm) and Lateral Left (LL), Lateral Right (LR), and Protrusive (PR) mandibular movements were also measured.

In addition, in the MP group, the mean level of pain experienced during the previous week was collected on a Visual Analogical Scale (VAS) ranging from 0 (No pain) to 10 (Extreme pain).

#### Psychological evaluation

The psychological evaluation included the assessment of psychological distress symptoms (depression, anxiety, and emotional distress), anger, and alexithymia.

### Psychological distress

Psychological distress—depressive, anxiety, and emotional distress symptoms—was evaluated by means of the following self-report scales.

#### Beck depression inventory—short form (BDI-SF)

The BDI-SF is a self–report measure composed of 13 items, rated on a 4-point scale ranging from 0 to 3, that assess the presence of depressive symptoms (Beck and Beck, 1972). It has an internal consistency level (coefficient alphas) comparable to that of the long form (Beck et al., 1988). The Pearson product-moment correlation coefficient between the BDI and the BDI-SF ranges from 0.89 to 0.97, indicating that the short form is an acceptable substitute for the long one (Beck et al., 1974).

#### State-trait anxiety inventory form Y (STAI-Y)

The State-Trait Anxiety Inventory Form Y (STAI-Y) differentiates between temporary or emotional state anxiety and long standing personality trait anxiety in adults (Spielberger et al., 1970). The STAI-Y is divided into two sections, each composed of twenty four-point Likert items: STAI-Y1 assesses state anxiety while STAI-Y2 assesses trait anxiety. The Italian version of the STAI-Y has shown a good internal consistency (Cronbach's alpha coefficient ranges from 0.91 to 0.95 for the Y1 subscale and from 0.85 to 0.90 for the Y2 subscale) and a good test-retest reliability (0.49 for the Y1 and 0.82 for the Y2 subscale) (Spielberger, 1989).

#### Distress thermometer (DT)

The Distress Thermometer (DT) is a simple checklist assessment tool commonly used in cancer care and other areas of physical health, such as chronic pain (Jacobsen et al., 2005). It is a valid rapid-screening instrument that measures the mean level of emotional distress experienced in the previous week on a 0–10 visual analog scale, in the form of a thermometer labeled with “No Distress” at 0, “Moderate Distress” at the midpoint and “Extreme Distress” at 10 (Grassi et al., 2013).

### Anger

#### State-trait Anger Expression Inventory—2 (STAXI-2)

The State-Trait Anger Expression Inventory-2 (STAXI-2) is a self-report scale composed of 57-items on a 4-point scale. It measures the intensity of anger experienced at a particular moment and the frequency with which the subject experiences, expresses, and controls feelings of anger (Spielberger, 1999). The instrument is composed of six scales, five subscales, and an Anger Expression Index. The Italian version has shown good validity and internal consistency (Cronbach's alpha coefficient ranges from 0.74 to 0.95) (Comunian, 2004).

### Alexithymia

#### Toronto Alexithymia Scale (TAS-20)

The Toronto Alexithymia Scale (TAS) is a self-report scale consisting of 20 items rated on a 5-point Likert scale (Bagby et al., 1994a,b) with a total score ranging between 20 and 100. According to the total score, the subject can be classified as non-alexithymic (score equal to or less than 51), borderline (score between 52 and 60) and alexithymic (score equal to or larger than 61).

The total score of the TAS-20 can be divided into three subscales: the Difficulty Identifying Feelings subscale (F1), the Difficulty Describing Feeling subscale (F2), and the Externally-Oriented Thinking subscale (F3). The Italian version of the TAS-20 has demonstrated factorial validity, a good internal consistency (Alpha = 0.75 in the normal sample and 0.082 in the clinical sample), and a high test-retest reliability over two-weeks (0.86 for the full scale) (Bressi et al., 1996).

### Statistical analysis

The data were analyzed using the SPSS-17 software package. Observed frequencies and percentages were used to describe categorical variables, whereas means and standard deviations were used for quantitative variables. Two independent sample t-tests were used to compare demographic, clinical, and psychological variables between the MP and the control groups. Pearson bivariate correlations were used to analyze the relationship between clinical and psychological variables in the MP group. P-values lower than 0.05 were considered statistically significant. Bonferroni correction was been applied for correlation analysis.

## Results

### Comparisons between the MP patients and the control group

#### Demographic and clinical features

The data regarding the demographic and clinical characteristics are presented in Table 1. The patients and control group did not significantly differ in age and educational level. Active and passive mouth opening measures were significantly different between the MP and C groups; the patients presented lower size movements than the controls. The PTS and CTS scores of the two groups indicated that muscle tenderness was significantly higher in the patients than in the controls.

#### Psychological distress

The results are presented in Table 2. Overall, the MP patients evidenced significantly higher psychological distress than the C group. Depression, state and trait anxiety and emotional distress scores (BDI, STAY-Y1, and –Y2, DT) were significantly higher in the MP patients.

**Table 2:** **Psychological distress in myofascial pain (MP) group and control group**.

	**MP group**	**Control group**	***T* (*df*)**	***P*-value**
BDI	6.7 (5.2)	2.5 (2.9)	4.82 (68.7)	<0.001
STAI-Y1	39.9 (10.9)	31.1 (6.4)	4.69 (71.1)	<0.001
STAI-Y2	45 (11.7)	35.1 (9.4)	4.39 (88)	<0.001
DT	4.7 (2.9)	2.7 (2.7)	3.3 (87)	0.001

Results of the T-test, used to compare psychological distress between MP patients and controls. BDI, beck depression inventory; STAI-Y1 and STAI-Y2, state-trait anxiety inventory form Y (1–2); DT, distress thermometer.

#### Anger

The data regarding the scales and subscales scores of the STAXI-2 are listed in Table 3. The only statistically significant difference between the two groups was found for “Anger Expression-In”: the MP patients showed a significantly higher tendency to suppress angry feelings.

**Table 3:** **Anger data of the myofascial pain (MP) group and the control group**.

	**MP group**	**Control group**	***T* (*df*)**	***P***
State anger	46.9 (9.1)	44.8 (6.4)	1.23 (88)	0.221
*Feeling angry*	47.4 (9.6)	45.1 (6.4)	1.34 (88)	0.183
*Feel like expressing anger verbally*	46.0 (6.1)	45.2 (5.1)	0.67 (88)	0.502
*Feel like expressing anger physically*	49.5 (11.5)	47.4 (6.5)	1.08 (70)	0.282
Trait anger	42.9 (10)	44.2 (9.1)	−0.66 (88)	0.509
*Angry temperament*	46.6 (9.7)	45.1 (8.1)	0.78 (88)	0.440
*Angry reaction*	45.1 (9.9)	44.8 (9.6)	0.11 (88)	0.914
Anger expression-out	42.3 (8.3)	43.2 (9.5)	−0.47 (88)	0.638
Anger expression-in	52.5 (10.9)	45.5 (10.8)	3.07 (88)	0.003
Anger control-out	49.2 (8.7)	51.7 (10.2)	−1.27 (88)	0.207
Anger control-in	52 (9.5)	55.6 (9.6)	−1.76 (88)	0.081
Anger expression index	45.6 (10.8)	42.7 (9.4)	1.38 (88)	0.173

Results of the T-test used to compare data of the scales and subscales of the STAXI-2 (State-Trait Anger Expression Inventory–2) between MP patients and controls.

#### Alexithymia

The data regarding alexithymia are shown in Table 4. The T-tests showed that the TAS-20 total score was significantly higher in the MP group with respect to the C group. As far as the three TAS-20 subscales are concerned, the MP patients showed significantly higher scores, compared to the C group, only in the subscales F1 (Difficulty in identifying feelings). With regard to alexithymia prevalence based on the TAS-20 cut-off: in the control group, 1 patient out of 45 (2.2%) was alexithymic, 7 out of 45 (15.6%) were borderline and 37 out of 45 (82.2%) non-alexithymic, while in the MP group, 4 patients out of 45 (8.9%) were alexithymic, 10 out of 45 (22.2%) borderline and 31 out of 45 (68.9%) non-alexithymic.

**Table 4:** **Alexithymia data of the myofascial pain (MP) group and the control group**.

	**MP group mean (*SD*)**	**Control group mean (*SD*)**	***T* (*df*)**	***P***
TAS-20	47.49 (10.6)	39.87 (11.1)	3.34 (88)	0.001
F1	17.71 (6.2)	12.73 (5.5)	4.05 (88)	<0.001
F2	13.53 (5.2)	11.49 (5.4)	1.84 (88)	0.07
F3	16.58 (4.4)	15.98 (4.8)	0.62 (88)	0.54

Results of the T-test, used to compare alexithymia between MP patients and controls. Abbreviations: TAS-20, toronto alexithymia scale; F1, difficulty identifying feelings subscale; F2, difficulty describing feelings subscale; F3, externally-oriented thinking subscale.

### Correlations between variables in the MP patients

#### Correlations between anger, alexithymia, and psychological distress

Correlation analysis was run only with variables showing a significant difference between the MP and the C group. These analyses are detailed in Table 5. Applying Bonferroni correction, correlations were considered significant for *p* < 0.017. The tendency to suppress angry feelings (Anger Expression-In score) showed a significant positive correlation with both depression score (BDI) and state and trait anxiety scores (STAI- Y1 and Y2), whereas no significant correlation was found with the emotional distress level (DT). In addition, Anger Expression-In scores showed a significant positive correlation with alexithymia (TAS-20 total score).

**Table 5:** **Correlations among Alexithymia, Anger Expression-In scores, and Psychological distress scales**.

	**BDI**	**DT**	**STAI-Y1**	**STAI-Y2**	**ANGER ER-IN**
ANGER ER-IN	0.466^**^	0.066	0.460^**^	0.464^**^	
TAS-20	0.348	0.083	0.570^**^	0.484^**^	0.406^*^
TAS-F1	0.330	0.242	0.478^**^	0.539^**^	0.244

Pearson bivariate correlations among psychological variables data in MP patients. Abbreviations: BDI, beck depression inventory; DT, distress thermometer; STAI-Y1 and STAI-Y2, state-trait anxiety inventory form Y (1–2); ANGER ER-IN, anger expression-inside; TAS-20, toronto alexithymia scale; TAS-F1, difficulty identifying feelings subscale.

* p < 0.017,

** p ≤ 0.001.

The TAS-20 total score and the F1 factor were significantly positively correlated with state and trait anxiety scores (STAI- Y1 and Y2). No significant correlations were found between alexithymia and the emotional distress, and between alexithymia and depression (the correlations did not reach the significant level after applying the Bonferroni correction).

#### Correlations between clinical and psychological variables

The correlation between the clinical indexes and psychological scales are shown in Table 6. Applying the Bonferroni correction, no significant correlation emerged between the data.

**Table 6:** **Correlations between clinical and psychological scales scores**.

	**BDI**	**TAS-20**	**TAS-F1**	**DT**	**ANGER ER-IN**	**STAI-Y1**	**STAI-Y2**
AO	0.01	−0.048	−0.088	−0.213	−0.206	−0.162	−0.142
PO	0.047	−0.017	0.036	−0.166	−0.149	−0.095	−0.087
PTS	−0.021	0.056	0.022	0.323	−0.197	−0.062	0.004
CTS	0.023	0.144	0.14	0.195	−0.139	−0.034	−0.085
VAS	0.051	−0.08	−0.085	0.119	−0.04	−0.09	0.034

Pearson bivariate correlations between clinical and psychological variables data in MP patients. Abbreviations: AO, active mouth opening; PO, passive mouth opening; PTS, pericranial tenderness score; CTS, cranial tenderness score; VAS, visual analog scale; BDI, beck depression inventory; TAS-20, toronto alexithymia scale; TAS-F1, difficulty identifying feelings subscale; DT, distress thermometer; ANGER ER-IN, anger expression-inside; STAI-Y1 and STAI-Y2, state-trait anxiety inventory form Y (1–2).

## Discussion

The aim of this study was to compare psychological distress, anger and alexithymia between a group of myofascial facial pain patients and a control group composed of healthy women, in order to identify the characteristic components of the MP patients. The relationships between psychological and clinical aspects were also analyzed.

The psychopathological characteristics associated with chronic pain pathologies have been widely investigated, but few studies have focused on MPS and most of them focused only on depression and anxiety.

Although the close relationship between chronic pain and depression is well documented (Torta and Munari, 2010), depression is often under-recognized and under-treated in patients with chronic muscle pain conditions (Greden, 2009). In agreement with the literature that highlighted a high prevalence of depressive symptoms in MPS (Celić et al., 2006; Altindag et al., 2008; Giannakopoulos et al., 2010), results of our study show a higher presence of depressive symptoms in the MP patients than in the healthy controls.

While the presence of depression in MPS is already well known, the role of anxiety is still under debate. Its role has been investigated in only a few studies, most of which analysed the presence of anxiety in TMDs, with contrasting results (Kino et al., 2001; Manfredini et al., 2004; Mongini et al., 2007; Giannakopoulos et al., 2010). Using a specific tool for assessing anxiety symptoms, the STAI-Y, we found that the MP patients were much more anxious than the healthy controls. Our result agrees with a previous study carried out by Vedolin, which found a higher level of anxiety in a group of MP patients compared to healthy controls (Vedolin et al., 2009).

Another commonplace feature in chronic pain patients is anger. Studying the presence, intensity, expression modalities and targets of anger may be important for understanding psychological adaptation to chronic pain (Okifuji et al., 1999). In a recent review, Trost analyzed the cognitive dimensions of anger in chronic pain and highlighted the importance of improving anger interventions in pain sufferers (Trost et al., 2012). Higher levels of both anger-out and anger-in management style are, in fact, associated with increased acute pain responsiveness and greater chronic pain intensity, although the mechanisms underlying the pain-exacerbating effects remain unclear (Janssen et al., 2001; Bruehl et al., 2002, 2003). The results of our study show that the MP patients did not differ from healthy controls with regard to trait or state anger. The only difference between the two groups was found on the Anger Expression-In scale, suggesting that the MP patients cope with emotions and physical or psychological distress by holding in or suppressing angry feelings. An adaptive way of showing anger feelings is to grit one's teeth and contract the neck muscles. One possibility is that by not expressing their anger feelings, MP patients keep them inside and develop a muscle contracture. In addition, previous evidence suggested that anger is related with both anxiety (Moscovitch et al., 2008; Hawkins and Cougle, 2011; Deschênes et al., 2012) and depression (Koh et al., 2002, 2008). This relationship was confirmed in our study, too. The Anger Expression-In scale was, in fact, positively correlated with depressive and anxiety symptoms: the higher the tendency to suppress anger, the higher the depressive and anxiety symptomatology.

Alexithymia is commonly present in people with psychosomatic or psychiatric disorders and is highly prevalent in chronic non-malignant pain patients (Lumley et al., 1997, 2002). The association between alexithymia and chronic pain in female patients has been pointed out by some studies (Sayar et al., 2004; Celikel and Saatcioglu, 2006; Tuzer et al., 2011), showing that alexithymia was more present in chronic pain patients than free pain controls (Celikel and Saatcioglu, 2006). In agreement with a previous study of Lumley et al. (2002), the data of our study showed that also the MP patients presented a higher level of alexithymia than the control group, especially with regard to difficulties in identifying feelings. Although alexithymia is considered a vulnerability factor for mental illness, it is still under debate whether it is associated with the development of specific mental disorders. Recently, Leweke found that alexithymia was strongly associated with depressive and anxiety disorders (Leweke et al., 2012). Our results showed that alexithymia was related to anxiety (the higher alexithymic traits, the higher the anxiety symptoms), but we did not find any correlation between alexithymia and depressive symptoms. A relationship emerged, instead, between Anger Expression-In and alexithymia: higher alexithymic traits positively correlated with higher internalized anger expression. The relationship between internalized anger expression and the alexithymic train could suggest a general inability of patients to recognize and manage their own emotions, which make it tough for them to access their internal states and communicate them to others. These disturbances could be a source of frustration, increasing anxiety and psychological distress and feeding the vicious circle of depression-pain-depression.

It has been speculated that the characteristics of alexithymic individuals seem to reflect the type D personality profile, which is characterized by high tendency to experience negative emotions and to inhibit the expression of emotions/behaviors in social interactions (Ogrodniczuk et al., 2012). Such as alexithymia, type D personality has been associated with poor physical and mental health outcomes, in particular in cardiovascular patients, in which type D personality has been linked to cardiovascular reactivity to acute stress. It has been shown that the tendency not to share negative feelings resulting from stressful emotional events is associated with exaggerated sympathetic and cardiovascular responses to later events, increasing the risk of develop cardiovascular disease like Stress Cardiomyopathy or Takotsubo cardiomyopathy (Compare et al., 2011, 2013). Taking into account these observations and the high presence of alexithymia and expression-in modality to cope with anger in MP patients, it would be interesting to analyze, in future researches, the possible relationship between type D personality profile and MP.

In contrast with previous study, we did not find any correlation between the clinical and the psychological parameters, possible due to methodological issues. In particular, the PTS and the CTS index have a very restricted range that could strongly influence the correlation's results. Mongini et al. (2007) found that the presence of anxiety and depression was positively associated with higher muscle tenderness scores. However, they used a cumulative tenderness score, given by the sum of the PTS and of the CTS, which could have bypassed this methodological problem. In addition, another issue that could have influenced the lack of correlation between the clinical and the psychological parameters is the relatively small sample size. In a larger sample, Altindag et al. (2008) found a significant positive correlation between depression and VAS pain score.

In conclusion, our results showed a high presence of psychopathology in the MP patients. Going further than previous studies, as well as confirming the high prevalence of depressive symptoms, our results suggest that MP patients are significantly more anxious, have a higher prevalence of alexithymia and an higher expression-in modality to cope with anger than healthy matched controls. Taken as a whole, the results of our case-control study highlight the need to include an accurate and specific psychological assessment of patients with MP in order to identify those psychological aspects, such as alexithymia and emotion management style, that could contribute to generate or exacerbate the suffering of these patients. It has, in fact, been suggested that the inability of individuals with alexithymia to adequately identify physical sensations, such as the somatic manifestations of emotions, makes it likely that they will incorrectly attribute innocent physical symptoms to physical disease (Tuzer et al., 2011). Including an accurate investigation of psychological aspects in MP patients in normal clinical practice would allow clinicians to screen and diagnose psychopathological features and to carry out more efficacious management strategies, such as psychopharmacological and especially psychotherapeutic interventions, i.e., cognitive-behavioral therapy or brief psychodynamic psychotherapy.

## Limitations

The main limitations of this study are the relatively small number of patients and the fact that the patients were enrolled from a single center. Further multicenter studies including a higher number of patients should be carried out in order to confirm these results and better understand the relationship between psychological symptoms and pain in MP patients.

### Conflict of interest statement

The authors declare that the research was conducted in the absence of any commercial or financial relationships that could be construed as a potential conflict of interest.
